# The marital and fertility sentiment orientation of Chinese women and its influencing factors – An analysis based on natural language processing

**DOI:** 10.1371/journal.pone.0296910

**Published:** 2024-02-21

**Authors:** Yiqing He, Noor Eshah Tom Abdul Wahab, Haslina Muhamad, Darong Liu

**Affiliations:** 1 Department of Anthropology and Sociology, Faculty of Arts And Social Sciences, University of Malaya, Kuala Lumpur, Malaysia; 2 Department of Artificial Intelligence, Faculty of Computer Science and Information Technology, University of Malaya, Kuala Lumpur, Malaysia; The University of Lahore, PAKISTAN

## Abstract

**Background:**

With the evolution of China’s social structure and values, there has been a shift in attitudes towards marriage and fertility, with an increasing number of women holding diverse perspectives on these matters. In order to better comprehend the fundamental reasons behind these attitude changes and to provide a basis for targeted policymaking, this study employs natural language processing techniques to analyze the discourse of Chinese women.

**Methods:**

The study focused on analyzing 3,200 comments from Weibo, concentrating on six prominent topics linked to women’s marriage and fertility. These topics were treated as research cases. The research employed natural language processing techniques, such as sentiment orientation analysis, Word2Vec, and TextRank.

**Results:**

Firstly, the overall sentiment orientation of Chinese women toward marriage and fertility was largely pessimistic. Secondly, the factors contributing to this negative sentiment were categorized into four dimensions: social policies and rights protection, concerns related to parenting, values and beliefs associated with marriage and fertility, and family and societal culture.

**Conclusion:**

Based on these outcomes, the study proposed a range of mechanisms and pathways to enhance women’s sentiment orientation towards marriage and fertility. These mechanisms encompass safeguarding women and children’s rights, promoting parenting education, providing positive guidance on social media, and cultivating a diverse and inclusive social and cultural environment. The objective is to offer precise and comprehensive reference points for the formulation of policies that align more effectively with practical needs.

## Introduction

Marriage and fertility patterns in Chinese society are undergoing significant changes, transitioning from high to low marriage and fertility rates, with more and more people choosing to remain single and childless. According to data from China’s seventh national population census in 2020 (http://www.gov.cn/xinwen/2021-05/11/content_5605879.htm), the annual number of registered marriages decreased from 13.47 million in 2013 to 8.13 million in 2020. In the same year, the average age at first marriage in major cities reached 30, and the national average age at first marriage exceeded 27. Additionally, China’s total fertility rate (TFR) has fallen below the “low fertility trap” threshold of 1.5, standing at only 1.3, which is significantly below the level required for generational replacement (2.1). These trends pose a significant challenge for Chinese society, which is currently grappling with the problems of low birth rates and population ageing. Deciding whether to marry and have children is closely related to the marriage and fertility intentions of young individuals, which, in turn, are highly influenced by their sentiment orientation towards marriage and fertility [1–3]. Women’s sentiment orientation towards marriage and fertility is particularly important, as women constitute the main demographic group involved in both marriage and child-bearing. Therefore, investigating women’s emotional orientation towards marriage and fertility, along with its influencing factors, can aid in understanding and formulating appropriate marriage and fertility policies to enhance China’s fertility rate.

Indeed, in Chinese society, marriage and family are fundamental units that significantly impact people’s well-being and social stability [4]. Research conducted by Liu [5] on Chinese families revealed that the process of modernisation in China remains closely intertwined with family relationships. The concept of family serves as an entry point for exploring the overall characteristics, transformations, and specific manifestations of Chinese civilization. A culture and traditions centred on the family represent the “constants” and foundation of Chinese society. Within the traditional family system, individual interests are subordinate to those of the family. While women play significant roles in family life, they are positioned in a state of dependence and subordination within the patrilineal inheritance system. These dynamics shape their formal social standing and meaning within the societal structure. Additionally, under the influence of traditional Confucian culture, blood relations hold the highest priority in China’s hierarchical social structure [6, 7]. The gender, order, and number of children are among the most important criteria used to evaluate women’s status and value in China [8]. Women are primarily responsible for making choices regarding marriage and fertility and they bear the primary consequences of these choices. Although women’s status in China is continuously improving, and today they are generally given room for independent thinking and decision-making, cultural inertia has led to the perpetuation of certain outdated traditional concepts and customs. For example, women are still encouraged to marry early and have children soon after marriage. Marriage remains a perceived prerequisite for fertility, resulting in the formation of a societal value system in which fertility is considered an implicit responsibility accompanying marriage. Women have little agency within this value system [9]. By exerting pressure women through such family and societal practices as forced marriage and coercive fertility, which inhibit social development, women are compelled to make decisions regarding marriage and fertility. Consequently, these traditional family cultural beliefs and societal norms have become significant factors influencing women’s sentiment orientation towards marriage and fertility, leading many Chinese women to develop a fear of marriage and fertility.

In recent years, more and more Chinese women have chosen to marry later or remain unmarried. This may to some extent indicate increasing questioning of the significance of marriage and fertility. Furthermore, the voices of women advocating for “no marriage, no fertility for peace of mind” on the Internet reflect the inclination of some young women to detach their personal happiness and worth from traditional roles in marriage and fertility. This also signifies a shift in women’s values regarding marriage and fertility, with a greater emphasis on their individual development and subjective sense of happiness within marriage [10]. Drawing on Becker’s *A Treatise on the Family*, Li and Shen [8] discussed the multidimensional factors contributing to women’s decision to marry and have children later in life. They argued that women are increasingly focusing on self-management and improving their quality of life, which leads to a more rational and clear perspective on marriage and fertility. Women are more inclined to seek fulfilment and accomplishment in their careers and personal development, which in turn leads to more cautious and rational choices regarding marriage and fertility [11].

In addition to traditional family culture, social norms, and individualised development, which shape values surrounding marriage and fertility, women’s sentiment orientation towards marriage and fertility is closely related to other factors. Scholars have explored these influencing factors using various research methods. Wang and Wu [12] found that some Chinese women, due to their familial upbringing, had concerns about becoming like their mothers or grandmothers, independently raising children with an absent father, and experiencing maternal challenges. These child-rearing concerns weighed heavily on them and created pressure and anxiety regarding marriage and family responsibilities. Such concerns about child-rearing and maternal challenges have become significant factors influencing women’s sentiment orientation towards marriage and fertility [13].

Furthermore, many women face common difficulties in balancing family and career roles [14–16]. Fellegi [17] found through official statistical data and interviews that the primary challenge faced by diplomats is the conflict between work and family. Moreover, due to the existing "double burden" and the specific "maternal concept" attributed to a profound understanding of gender roles, it is evident that women are disproportionately affected. These obstacles originate at the individual, institutional, and national levels, which are closely interconnected and exhibit historical and political path dependencies. Lin and Xie [18], taking a life-course perspective, discovered that for some women with high career aspirations, pursuing career development led to the postponement of marriage and fertility or the decision not to have children altogether, resulting in a negative sentiment orientation towards marriage and childbearing. Ye’s [19] research indicated that many companies have implicit requirements and conditions linked to marriage and fertility when recruiting female employees, such as providing inadequate maternity leave or restricting promotions for female employees. These objective factors, which lead to reduced economic income or job loss due to marriage and fertility, have become the most common and prevalent factors influencing women’s attitudes and sentiment orientation towards marriage and fertility [20, 21]. Therefore, although women are the primary decision-makers regarding marriage and fertility, they are influenced by societal trends, family cultural beliefs, career aspirations, support for marriage and fertility, and concerns about motherhood. These factors covertly shape their sentiment orientation towards marriage and fertility. Consequently, contemporary Chinese women’s sentiment orientation towards marriage and fertility is the outcome of multifactorial influences.

Research on women’s sentiment orientation towards marriage and fertility has predominantly utilised methods such as questionnaire surveys [2], interviews [22], and case studies [23]. However, these methods have certain limitations, including subjectivity, respondent bias, and data quality issues. Therefore, addressing the limitations of traditional methods, such as questionnaire surveys, interviews, and case studies, in capturing the nuanced and dynamic nature of women’s sentiments on marriage and fertility has become an urgent issue.

As China’s marriage and fertility patterns continue to evolve, and the factors influencing women’s decisions become increasingly complex, natural language processing has emerged as a revolutionary method for analyzing large volumes of textual data [24, 25]. This approach provides a more comprehensive understanding of sentiment orientation, allowing for a more thorough exploration of women’s emotional tendencies regarding marriage and fertility and their influencing factors. The main contributions and innovations of this study are outlined as follows:

1. Introducing natural language processing as a research tool revolutionizes the study of women’s sentiments on marriage and fertility. This method overcomes biases and data quality issues inherent in traditional surveys, interviews, and case studies, enabling thorough analysis of vast textual datasets.
2. Natural language processing uncovers both explicit and subtle sentiments, offering profound insights into women’s attitudes toward marriage and fertility. This richer understanding helps policymakers align interventions with women’s genuine feelings and desires.
3. Incorporating natural language processing enhances policy formulation regarding marriage and fertility. By identifying sentiment trends among different groups, policies can be tailored to diverse perspectives, leading to more effective and sensitive interventions.
4. The automated sentiment analysis facilitated by natural language processing unveils latent influencing factors that shape women’s perspectives on marriage and fertility, thereby enriching the discourse within this domain.

In summary, this study, through advancements in research methodology using natural language processing, aims to provide a more accurate and nuanced description of women’s emotional orientations. By doing so, it seeks to offer a deeper understanding of the diversity and collective characteristics of marriage and fertility among Chinese women. The ultimate goal is to provide a more accurate and comprehensive reference basis for the formulation of policies that better align with the actual needs of the population, addressing the complexities of contemporary issues related to marriage and fertility in Chinese society.

## Empirical research on natural language processing based on sentiment analysis, Word2Vec, and TextRank techniques

### Research objectives and data sources

#### Research objectives

This study used natural language processing (NLP) techniques to explore and analyse the sentiment orientation of Chinese women towards marriage and fertility, as well as their influencing factors. The data were collected from Weibo, China’s foremost social media platform, in relation to “women’s marriage and fertility” hot relevant topics. The findings of the analysis provide effective guidance and recommendations for the government and society to promote the continuous development of marriage and fertility patterns in Chinese society.

#### Data sources

The study obtains data from user comments on the API interface of a public forum, excluding clinically relevant content. Ethical justification for data collection is explored from four perspectives. Privacy concerns are addressed by de-identifying user information to prevent significant privacy breaches. The principle of informed consent is upheld as the forum informs users during registration about data collection, use, and sharing, allowing them to provide informed consent when registering. The collection and utilization of data in this study are exclusively intended for this research, adhering to data protection laws, anonymizing sensitive information, and preventing misuse, without commercial purposes. Data will not be shared with third-party platforms and remains proprietary to this study.

We conducted a search in the “women’s marriage and fertility” section of popular topics on Weibo. We found six hot topics for discussion, labelled as “Prohibition on Inquiring about Women’s Marriage and Fertility in Job Interviews,” “The Impact of App Usage Habits on College Students’ Attitudes towards Marriage and Fertility,” “How to View the Increasing Trend of Women’s Marriage and Fertility Delay,” “Tendency of Some Young Women Towards Non-Marriage and Non-Childbearing,” “Why Are Women’s Topics Always Related to Marriage and Fertility?”, and “The Marriage and Fertility Dilemma of Contemporary Young Women.” These topics yielded a total of 3,200 discussion data points, consisting of over 30,000 words in qualitative data. The data were collected for analysis and exploration in this research.

### Measures

#### Sentiment analysis

The two most commonly used Chinese NLP tools are Tencent Cloud NLP and Baidu AI NLP (AI NLP). Tencent Cloud NLP has a maximum text length limit of 200 characters, while AI NLP has a limit of 1024 characters for analysis. As some of the comments collected from Weibo exceeded 200 characters, this study used AI NLP for analysis. AI NLP provides sentiment analysis functionality, utilising deep learning techniques and Baidu’s extensive data, to automatically determine the positive, neutral, or negative sentiment orientation of Chinese text and quantify its intensity. The tool outputs positive and negative sentiment scores that sum up to 1 [26].

#### Word2Vec

This study adopted Word to Vector (Word2Vec) as another NLP tool. Word vectors were initially proposed by Hinton [27], and Bengio et al. [28] established the earliest original model for word vectors. This method can be divided into two main types: one-hot representation and distributed representation. The former has a simple representation approach but limited semantic expression capability, while the latter is an advanced model based on the former, which to some extent compensates for the limited semantic expression capability and the problem of sparse and lengthy matrices [29]. Word2Vec is an NLP tool introduced by Google in 2013, and its internal algorithm draws on the basic concepts of neural network language model. Its advantage lies in mapping words in the text to a real-valued vector space based on a given corpus, where the real-valued vector space consists of multiple dimensions, each representing a corresponding shallow semantic feature [30]. Mature Word2Vec tools consist of two main models: CBOW and Skip-Gram. Due to the large size of the training dataset in this study, we used the Skip-Gram model, which has the characteristics of precise semantics and excellent performance in large training datasets, as it can measure contextually related words given a single word as input [31].

#### TextRank

TextRank is a natural language algorithm that is used for semantic analysis. Its predecessor was the PageRank algorithm developed by Google for web page ranking. TextRank transforms the natural language text to be analysed into a network graph and then ranks the weights of various nodes in the graph through analysis. This allows the user to obtain the ranking of important nodes [32], as the importance of a node depends on the number of neighboring nodes pointing to it. In TextRank, the importance of nodes changes iteratively based on the algorithm, so it is necessary to determine the convergence of node analysis to stop the iteration and establish the importance of nodes [33].

### Research process

**Fig 1:**
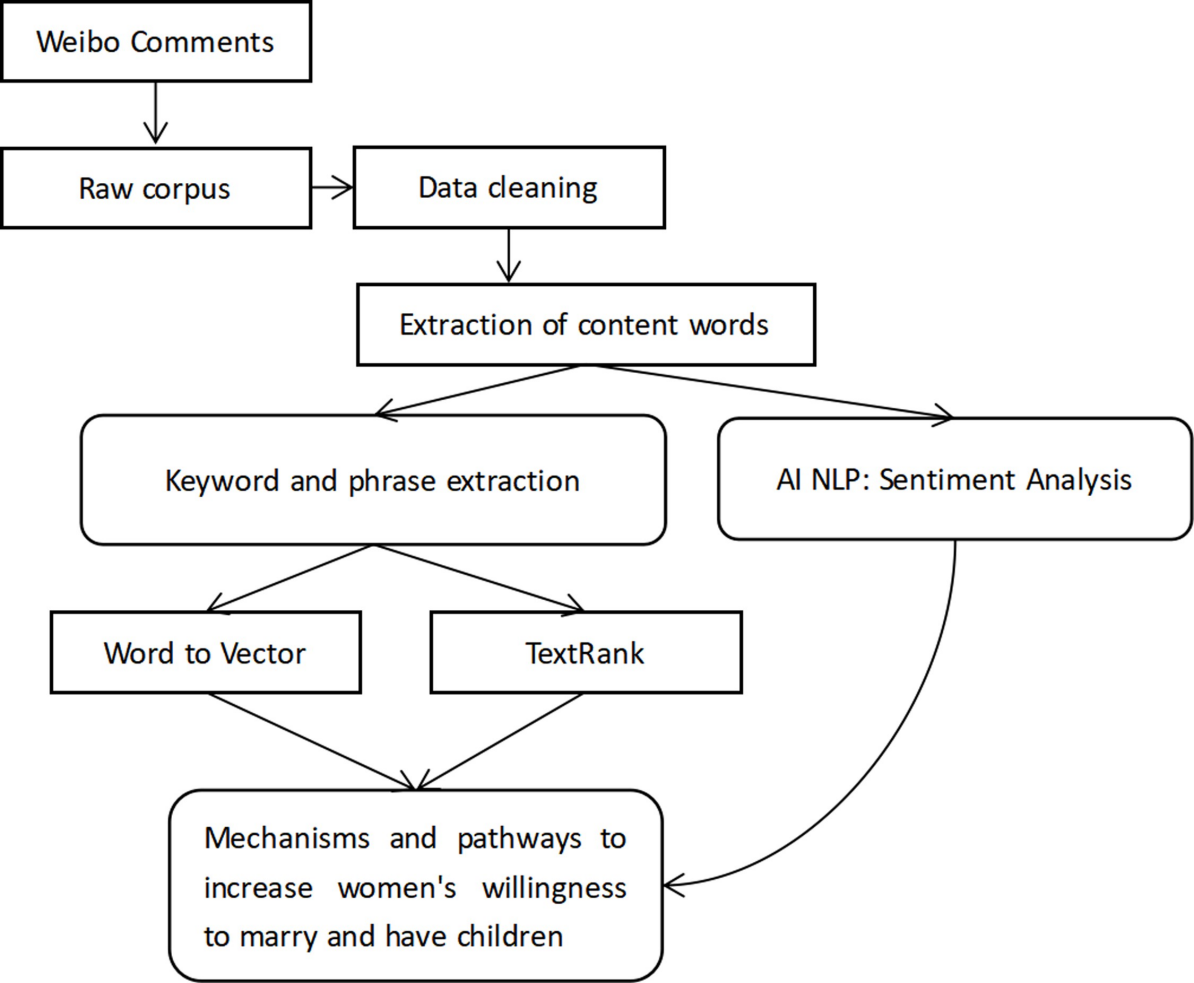
Research process diagram.

The research process consists of five main stages: (1) preprocessing of the raw corpus, (2) extraction of content words, (3) sentiment analysis, (4) extraction of key words and phrases using Word2Vec and TextRank, and (5) presentation of research findings (Fig 1).

This study used NLP techniques to analyse keywords and phrases. The specific content and processes were as follows.

#### Preprocessing of the raw corpus

Prior to conducting this study, the original corpus has been anonymized, and no identifiable information of the commenters is accessible. Due to the usage of Chinese text in this study, it was necessary to conduct relevant preprocessing of the original corpus before the computation began. Simultaneously, irrelevant stop words were removed to avoid corresponding noise interference and ultimately enhance the precision of the topic. Additionally, to improve the effectiveness of extracting content words in the later stages of the research, part-of-speech tagging was employed. The part-of-speech tagging tool used in this study was the NLPIR Chinese Word Segmentation System [34].

#### Extraction of content words

This study conducted a full-text retrieval of all relevant textual data with the aim of extracting nouns, verbs, adjectives, and adverbs from the text. We used a classical keyword weighting formula known as term frequency–inverse document frequency (TF-IDF). By calculating the TF-IDF values of the extracted vocabulary, we were able to filter out words with TF-IDF values below a specified threshold, resulting in the formation of a corresponding collection of content words. The TF-IDF calculation formula was as follows:

TF−IDF(t,d,D)=TF(t,d)×IDF(t,D), (1)

$$\text{TF}(t,d)=\frac{\text{Number}\;\text{of}\;\text{times}\;\text{word}\;t\;\text{appears}\;\text{in}\;\text{document}\;d}{\text{Total}\;\text{number}\;\text{of}\;\text{words}\;\text{in}\;\text{document}\;d},$$ (2)

$$\text{IDF}(t,D)=\text{log}\left(\frac{\text{Total}\;\text{number}\;\text{of}\;\text{documents}\;\text{in}\;\text{corpus}\;D}{\text{Number}\;\text{of}\;\text{documents}\;\text{in}\;\text{corpus}\;D\;\text{containing}\;\text{word}\;t+1}\right),$$ (3)

Note: *t* represents the target word. *d* represents the target document. *D* represents the entire corpus. The "+1" in the denominator of the IDF formula is added to avoid division by zero, which is a smoothing technique often referred to as "add-one smoothing."

#### Sentiment analysis

To capture topics in Weibo comments related to Chinese women’s sentiment orientation towards marriage and fertility, Python 3.7.0 was used to install the Baidu AI NLP software development kit. The AI NLP interface was imported and the obtained Weibo text data were inputted. The sentiment analysis function within the toolkit was utilised to obtain positive and negative sentiment scores for each comment. Comments with a confidence score below 0.8 were excluded, as they were considered to be insufficiently relevant to the topic.

#### Extraction of key words using Word2Vec

1. Obtaining real-word vectors based on Word2Vec technology. We retrieved relevant hot topics related to women’s marriage and childbirth from Weibo. Each hot topic exhibited a certain degree of relevance and thematic coherence, and the usage context of terms was also relatively similar. Therefore, determining the semantic meaning and content based on the contextual information of the words can yield accurate results.
2. Obtaining topic words and their vector representations through keyword vector clustering. We employed the X-means algorithm, which is an improved version of the K-means algorithm, for keyword vector clustering. One advantage of this algorithm is that it does not require users to specify the number of clusters K at the beginning. Instead, a range of values for K is defined, and the algorithm dynamically determines the optimal number of clusters within that range, resulting in an optimised clustering of keyword vectors.
3. Calculating the semantic similarity of topic words based on word vectors. The semantic similarity is determined by the cosine distance between two sets of real-word vectors. We used an improved version of the cosine similarity formula to calculate the semantic similarity between two topic words, C1 and C2. Assume that the topic word C1 has a set of words {W11, W12, …, W1m} and the set of words under the topic word C2 is {W21, W22, …, W2n}, where m > n. Then, the formula for calculating the cosine similarity is as follows:

$$\text{cosine\_similarity}(C1,C2)=\frac{\sum_{i=1}^{m}\sum_{j=1}^{n}\text{sim}(W1i,W2j)}{\sqrt{\sum_{i=1}^{m}\sum_{j=1}^{n}\text{sim}(W1i,W1i)}\cdot \sqrt{\sum_{i=1}^{m}\sum_{j=1}^{n}\text{sim}(W2j,W2j)}},$$ (4)

Note: *C*_1_ and *C*_2_ are the topic words being compared. *W*1*i* represents the *i*th word from the set of words under topic word *C*_1_. *W*2*i* represents the *j* th word from the set of words under topic word *C*2.

*m* and *n* are the number of words in the sets associated with *C*1 and *C*2, respectively.

sim(*W*1*i*, *W*2*j*) represents a measure of semantic similarity between words *W*1*i* and *W*2*i*.

#### Extraction of key phrases using TextRank

The logic of TextRank is based on the principle that the importance of a node depends on the number of neighbouring nodes that point to it. Let V be the set of nodes and E be the set of edges. *OD*(*v*_*i*_) represents the out-degree of node *v*_*i*_, where d is a damping factor ∈ [0,1]. The damping factor ensures that each node has a 1-d probability of random jumping, which helps the algorithm converge on any graph. Therefore, the TextRank value of node i, denoted as *T*(*i*), is calculated using the following formula:

$$T(i)=(1-d)+d\times \sum_{j\in \text{In}(i)}\frac{1}{\text{OD}(j)}\times T(j),$$ (5)

**Fig 2:**
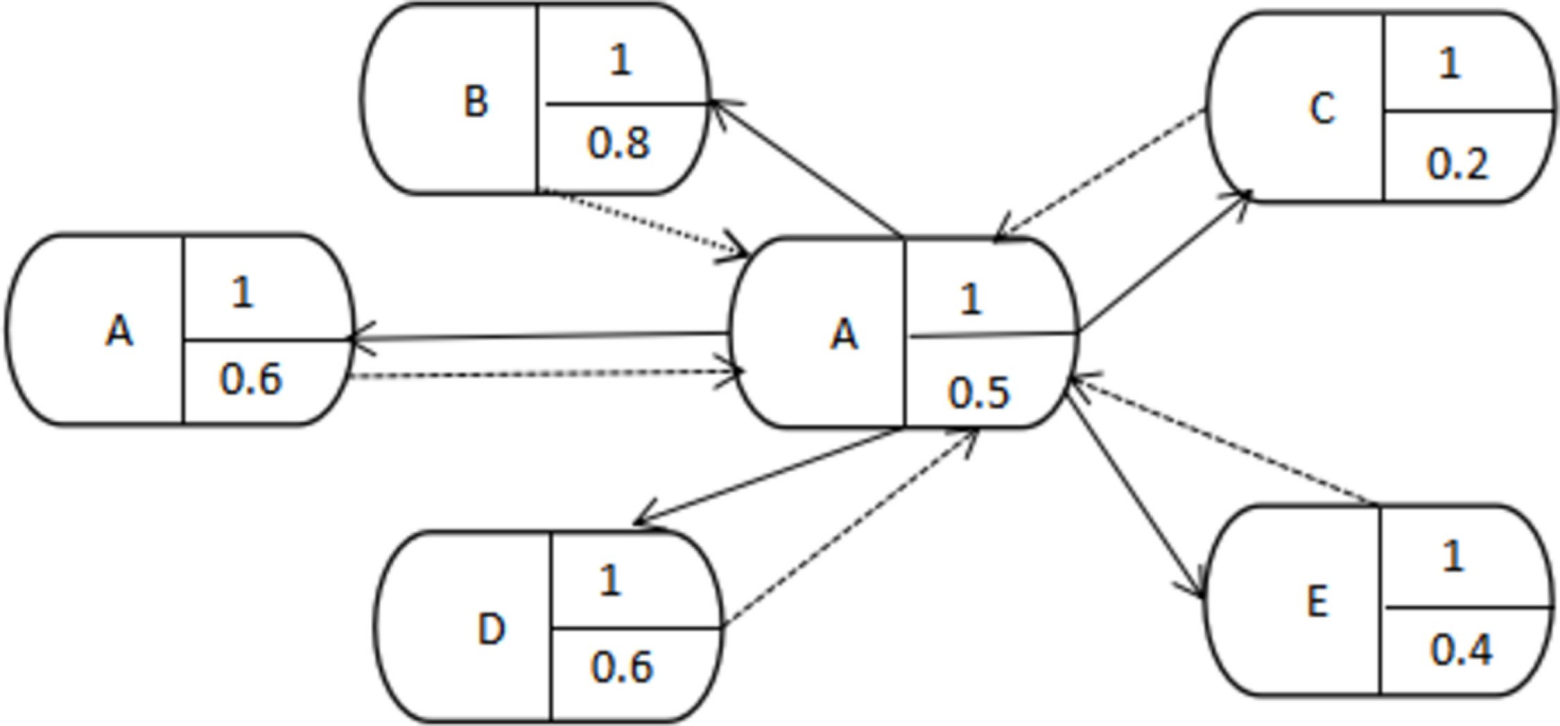
Example of TextRank candidate keywords graph illustration.

TextRank takes into account the thematic influence of words in the calculation process. The properties of a word’s graph node vi are divided into two parts. One is the importance value of the node in the internal structure of the text, which is represented by TR(vi) and has a default value of 1, and is iteratively updated based on the branches of adjacent nodes. The other part is the node’s own thematic influence value, which represents the external information mapped onto the node and is denoted as TI(vi). For example, the TextRank algorithm with six nodes (ADCDEF) has candidate words as shown in Fig 2.

The iteration of TextRank is a complex process that involves various aspects, such as iterative calculation based on the importance of new nodes, a probability transition matrix, and a topic influence matrix [35].

## Results

### Algorithm and pseudocode

This section mainly introduces the algorithm flow used in this paper, in the form of pseudo code.

**Algorithm:** Sentiment Analysis of Topics Related to Women’s Marriage and Fertility
Function baiduLSTMClassification(text):
 **Input:** The original text from the dataset.
 **Output:** Classification result
 // Invoke Baidu API’s LSTM model for sentiment classification
 result = BaiduAPICall(text, "LSTM")
 return result
Function word2vecTokenization(negativeText):
 **Input:** Negative emotional text
 **Output:** Word segmentation result
 // Apply Word2Vec algorithm for tokenizing the text
 tokens = Word2VecTokenize(negativeText)
 return tokens
Function wordCloudAnalysisAndVisualization(tokens):
 **Input:** Word segmentation result
 **Output:** Picture of visualization the WordCloud
 // Utilize WordCloud tool to generate a word cloud representation
 wordCloud = GenerateWordCloud(tokens)
 // Visualize the generated word cloud
 VisualizeWordCloud(wordCloud)
Function textrankSorting(tokens):
 **Input:** Word segmentation result
 **Output:** Sorted Result
 // Apply the TextRank algorithm to sort the tokenized words
 sortedTokens = TextRankSort(tokens)
 return sortedTokens
// Main Program
Function main():
 // Read the corpus
 corpus = ReadCorpus()
 // Perform sentiment classification on the corpus
 sentimentResult = baiduLSTMClassification(corpus)
 // Extract the text associated with negative sentiment
 negativeText = ExtractNegativeText(sentimentResult, corpus)
 // Tokenize the text associated with negative sentiment using Word2Vec
 negativeTokens = word2vecTokenization(negativeText)
 // Conduct word cloud analysis and visualization
 wordCloudAnalysisAndVisualization(negativeTokens)
 // Utilize TextRank for sorting the tokenized words
 sortedTokens = textrankSorting(negativeTokens)
 // Output the sorted tokens
 OutputSortedTokens(sortedTokens)
// Invoke the main program
main()

### Sentiment analysis of topics related to women’s marriage and fertility

#### Explanation of credibility in Baidu AI NLP

This study utilises the PaddlePaddle framework developed by Baidu in China to construct a bidirectional long–short-term memory (LSTM) network. This network efficiently and accurately analyses the sentiment embedded in text and determines the sentiment polarity, thus classifying sentiment orientation [30]. Unlike simple recurrent neural networks, LSTM incorporates memory cells (c), input gates (i), forget gates (f), and output gates (o). These gates and memory cells greatly enhance the ability of recurrent neural networks to process long sequential data. In the process of training recurrent neural networks, the issue of vanishing or exploding gradients often arises when dealing with longer sequence data. LSTM was proposed by Hochreiter and Schmidhuber in 1997 to address this problem [36]. By introducing memory and control gates to simple recurrent neural networks, LSTM strengthens its ability to handle long-term dependencies. A similar improvement is represented by gated recurrent units (GRUs) [37], which have a more concise design. Although these improvements have their own distinctive characteristics, their macro descriptions are similar to those of simple recurrent neural networks, where the hidden state changes based on the current input and the previous hidden state, continuously iterating this process until the input is fully processed. If the function represented by the LSTM-based recurrent neural network is denoted as F, the formula is as follows: *h*_*t*_ = *F*(*X*_*t*_, *h*_*t*-1_), where F is composed of the following equations:

$$i_{t}=\sigma \left(W_{xi}X_{t}+W_{hi}h_{t-1}+b_{i}\right),$$ (6)

$$f_{t}=\sigma \left(W_{xf}X_{t}+W_{hf}h_{t-1}+b_{f}\right),$$ (7)

$$g_{t}=\tanh\left(W_{xg}X_{t}+W_{hg}h_{t-1}+b_{g}\right),$$ (8)

$$o_{t}=\sigma \left(W_{xo}X_{t}+W_{ho}h_{t-1}+b_{o}\right),$$ (9)

$$c_{t}=f_{t}⊙c_{t-1}+i_{t}⊙g_{t},$$ (10)

$$h_{t}=o_{t}⊙\tanh\left(c_{t}\right),$$ (11)

Note: *X*_*t*_ is the input at time step *t*. *h*_*t*-1_ is the hidden state from the previous time step. *i*_*t*_,*f*_*t*_,*g*_*t*_,*o*_*t*_, are the input, forget, gate, and output gates’ activations respectively. *c*_*t*_ is the cell state at time step *t*. *σ* represents the sigmoid activation function. tanh is the hyperbolic tangent activation function. *W* matrices and *b* vectors are weight parameters and biases for different gates.

In this study’s experiment, sentiment prediction was performed on comments related to women’s marriage and fertility. First, word embeddings were computed using Embedding. Next, features were extracted and fused using bidirectional LSTM. A classifier was built with the help of the softmax function to obtain the sentiment inclination of the text information. The experimental results were quite promising.

#### Analysis of women’s sentiment orientation towards marriage and fertility for different topics

**Fig 3:**
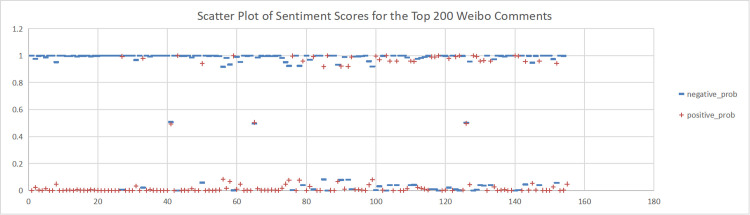
Scatter plot of sentiment scores for the top 200 Weibo comments. (A)+positive_prob. (B) -negative_prob.

To explore Chinese women’s current sentiment orientation towards marriage and fertility and its influencing factors, this study took over 3,200 comments from six hot topics relating to women’s marriage and fertility on Weibo as case studies. We used NLP techniques such as sentiment analysis, Word2Vec, and TextRank. First, we used the AI NLP tool to calculate the sentiment scores for these comments. For each topic, we set the sum of positive and negative sentiment scores to 1. Next, we cleaned the data by removing stop words and comments with a confidence score below 0.8. We then analysed the sentiment scores for the top 200 comments, as shown in Fig 3. As shown in the figure, among these six topics, comments related to women’s marriage and fertility expressed predominantly negative sentiment, with fewer instances of positive sentiment. Therefore, we inferred that the overall sentiment orientation towards women’s marriage and fertility on Weibo was negative, indicating predominantly negative attitudes among Chinese women towards marriage and fertility.

**Fig 4:**
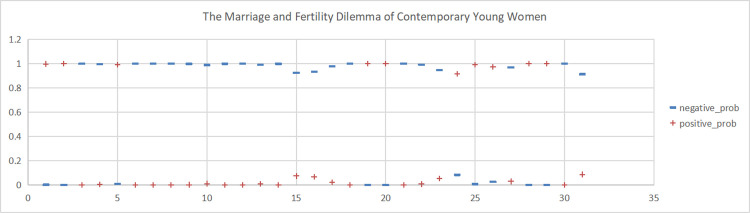
Sentiment scores for the Weibo topic "The Marriage and Fertility Dilemma of Contemporary Young Women”. (A)+positive_prob. (B) -negative_prob.

**Fig 5:**
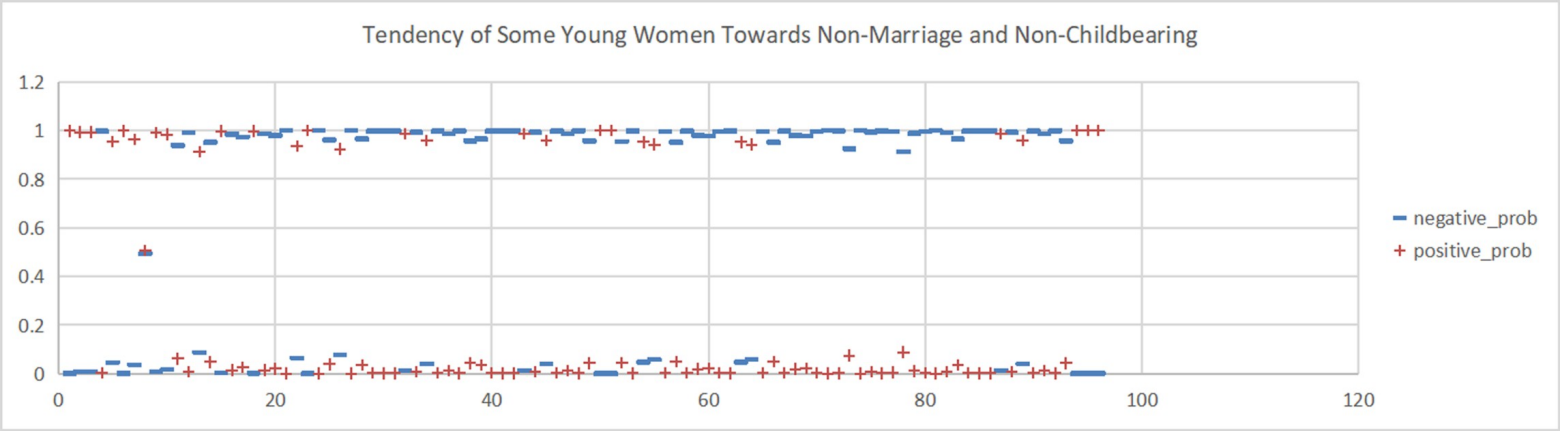
Sentiment scores for the Weibo topic "Tendency of Some Young Women Towards Non-Marriage and Non-Childbearing". (A)+positive_prob. (B) -negative_prob.

**Fig 6:**
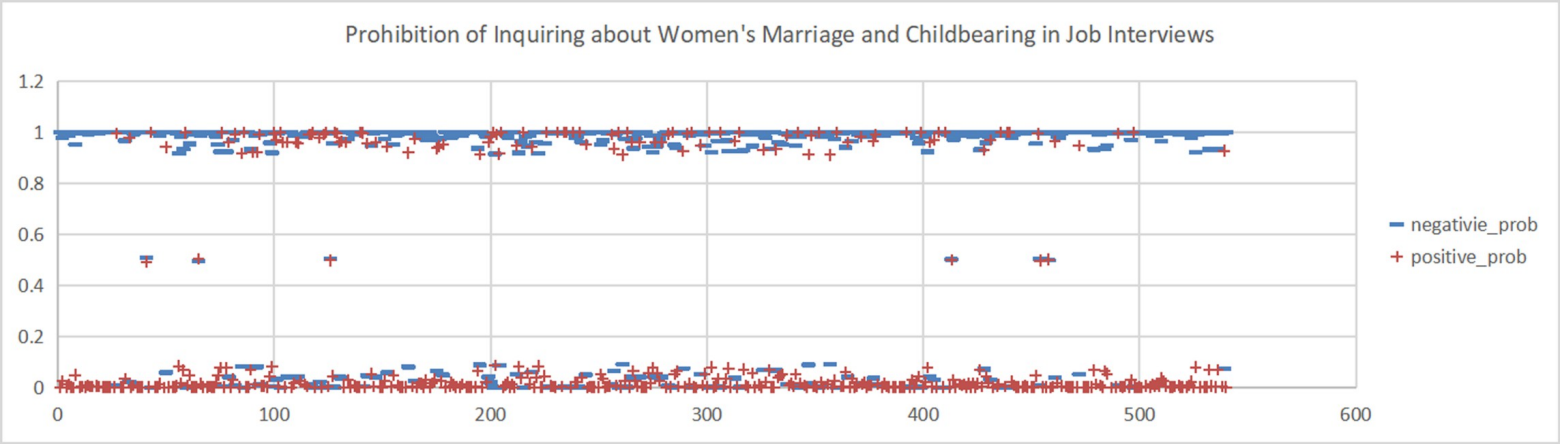
Sentiment scores for the Weibo topic "Prohibition of Inquiring about Women’s Marriage and Fertility in Job Interviews". (A)+positive_prob. (B) -negative_prob.

**Fig 7:**
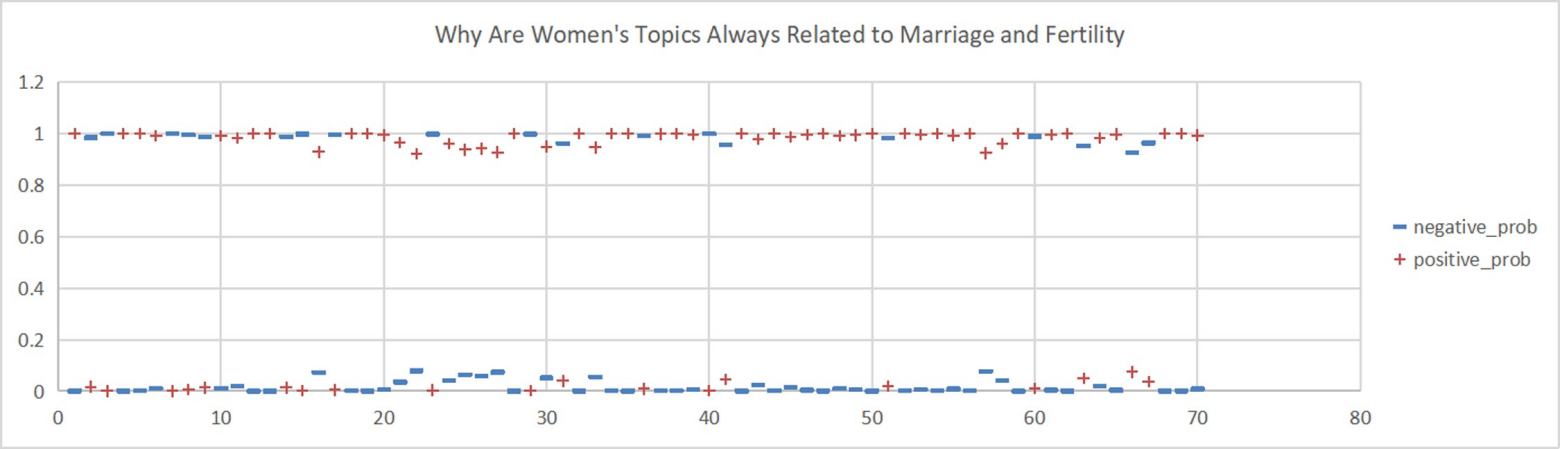
Sentiment scores for the Weibo topic "Why Are Women’s Topics Always Related to Marriage and Fertility". (A)+positive_prob. (B) -negative_prob.

**Fig 8:**
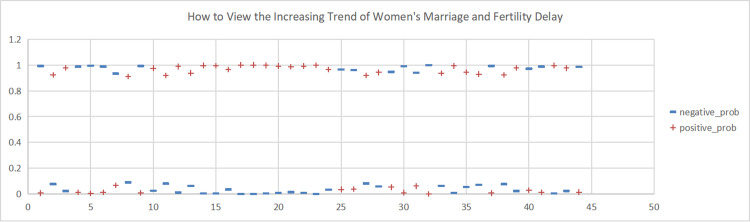
Sentiment scores for the Weibo topic "How to View the Increasing Trend of Women’s Marriage and Fertility Delay". (A)+positive_prob. (B) -negative_prob.

**Fig 9:**
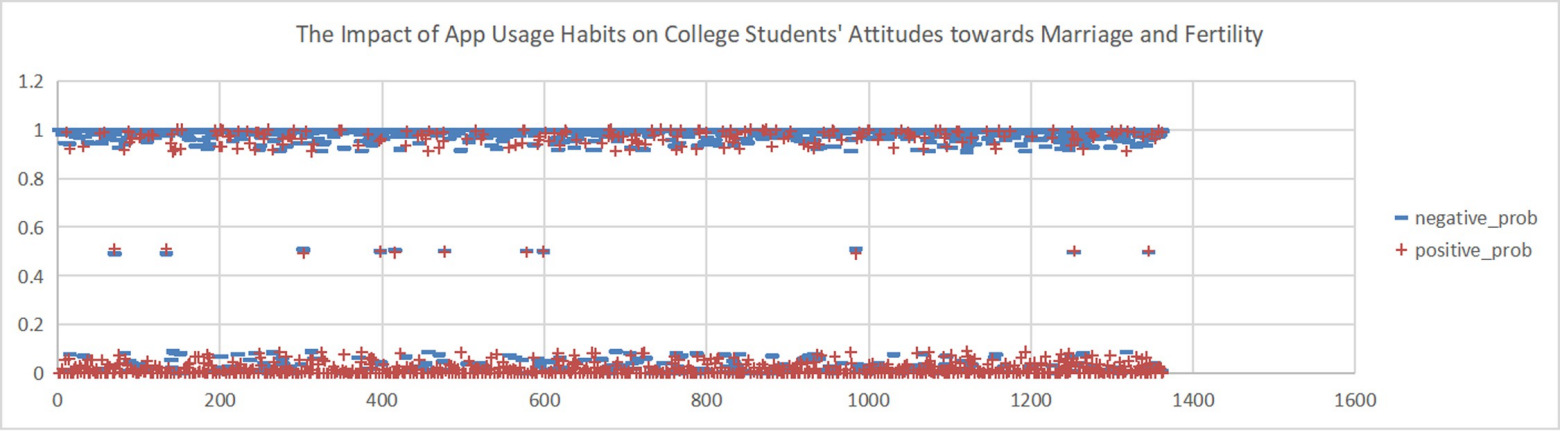
Sentiment scores for the Weibo topic "The Impact of App Usage Habits on College Students’ Attitudes towards Marriage and Fertility". (A)+positive_prob. (B) -negative_prob.

To analyse the sentiment orientation and sentiment scores of the six topics related to women’s marriage and fertility in a more detailed and specific manner, we conducted sentiment analysis for each of these six topics. This was done to accurately express the sentiment polarity of Weibo comments. For detailed information, please refer to Figs 4–9.

This study conducted sentiment analysis on six topics related to women’s marriage and fertility and obtained emotion scores for each topic. As shown in Figs 4, 5, 6, we found predominantly negative sentiment scores for comments regarding women’s marriage and fertility difficulties, the decreasing emphasis on marriage and fertility, and the issue of not inquiring about women’s marriage and fertility in job interviews. This suggests that women’s sentiment orientation towards marriage and fertility is negative and adverse. The data from Figs 7 and 8 demonstrate a higher occurrence of positive sentiment scores in comments discussing the association between women’s topics and marriage and fertility, as well as the increasing trend of delayed marriage and fertility. A possible reason for the higher positive sentiment scores in the discussion of the association between women’s topics and marriage and fertility could be the growing attention to and support for women’s rights and autonomy. People in China are recognising that women should have the right to pursue their own interests, and that marriage and fertility are just aspects of their lives. Thus, the higher positive sentiment scores for these topics may reflect support for these values. As for discussion of the increasing trend of delayed marriage and fertility, the higher positive sentiment scores may be attributable to an increasing awareness of the importance of gender equality in the workplace, education, and life. People are more inclined to express positive views on and support for delayed marriage and fertility, believing that this provides women with more opportunities and freedom to pursue their personal career and life goals. Fig 9, which reflects our exploration of the impact of app usage habits on college students’ views on marriage and fertility, also indicates a positive sentiment orientation in the comments. This may be because college students’ views on marriage and fertility are important indicators that influence future marriage and fertility rates. Given the significant influence of social media apps on college students, how app usage habits affect young people’s views on marriage and fertility is attracting increasing attention.

### Dimensions of and factors influencing negative sentiment orientation towards women’s marriage and fertility

#### Word cloud

As demonstrated in the previous section of this study, we found increasing attention to the topic of women’s marriage and fertility on Weibo, and the majority of the comments expressed a negative sentiment. Therefore, it was necessary to explore the factors that contributed to this negative sentiment towards women’s marriage and fertility. In light of this, this study utilised a word cloud to visually present the factors influencing negative sentiment towards women’s marriage and fertility.

**Fig 10:**
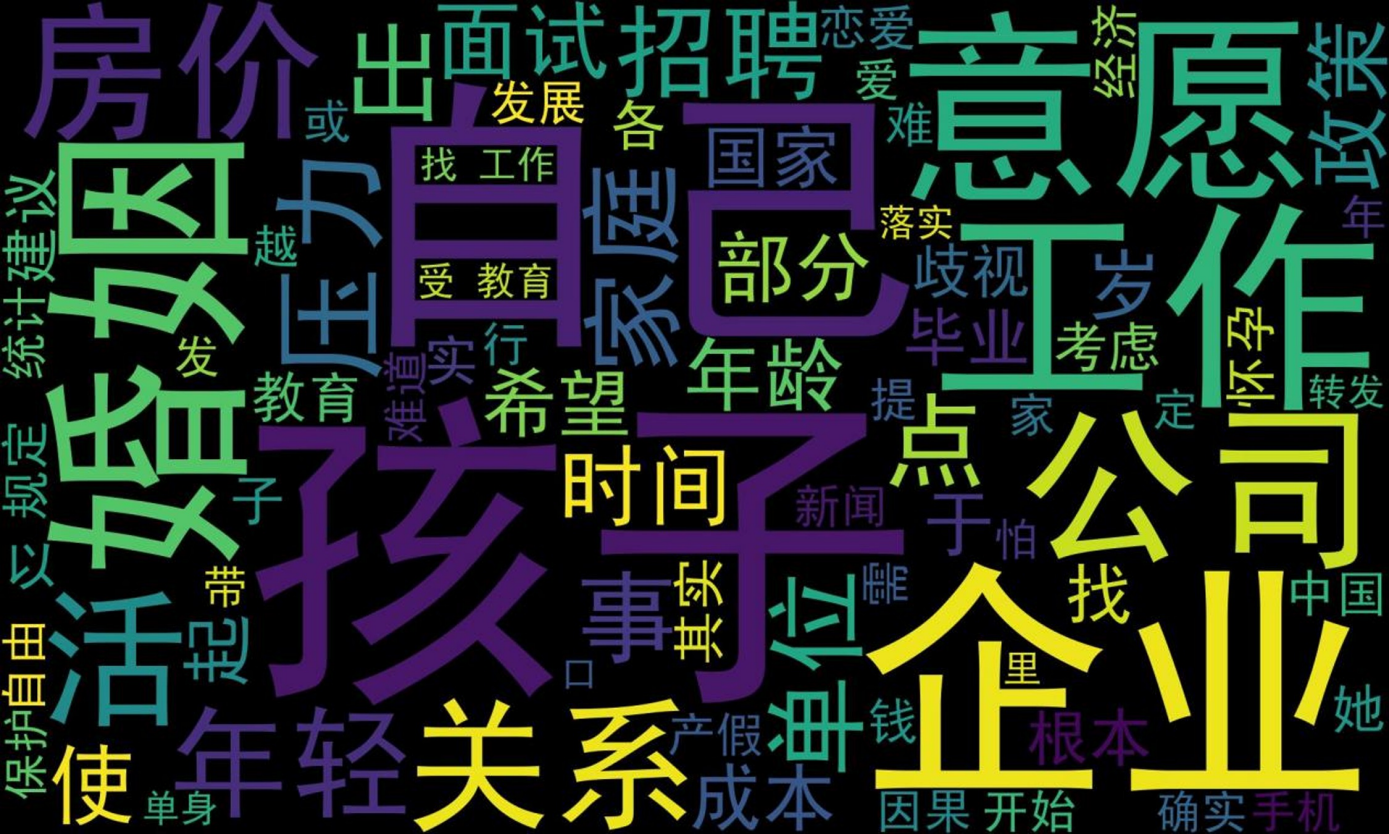
Word cloud of influencing factors for the negative sentiment towards women’s marriage and fertility.

In NLP, a word cloud is a visualisation tool used to display frequently occurring words in textual data [38, 39]. In a word cloud, the size and colour of the font typically represent the importance or frequency of a word in the text data. Larger font usually indicates a higher frequency of occurrence of that word in the text, while font colour is often used to represent the importance or emotional connotation of the word. For example, red may indicate positive or favourable sentiment, while blue may indicate negative or unfavourable sentiment. The word cloud of factors influencing the negative sentiment towards women’s marriage and fertility is shown in Fig 10, which helps us to visualise and analyse the key factors influencing the negative sentiment towards women’s marriage and fertility, facilitating subsequent weight ranking.

### Results of keyword extraction based on Word2Vec technology

By creating a word cloud, we were able to clearly observe the key factors influencing the negative sentiment orientation towards women’s marriage and fertility. To provide a clearer visualisation of the importance ranking of each keyword, we conducted keyword extraction using Word2Vec technology after processing a variety of textual content. The results, showing the top 10 ranked keywords, are presented in Table 1.

**Table 1:** Ranking of key words extracted based on Word2Vec technology.

Rank	Key words	Weight
1	孩子 Children	0.011149268476266641
2	自己 Myself	0.00875005880415863
3	企业 Enterprise	0.0068212824010914054
4	工作 Work	0.006350849132050618
5	意愿 Intention	0.005080679305640495
6	招聘 Recruitment	0.004704332690407866
7	婚姻 Marriage	0.004610246036599709
8	教育 Education	0.0037164228254222137
9	公司 Company	0.003669379498518135
10	房价 Housing Price	0.003669379498518135

#### Results of key phrase extraction based on TextRank technology

After showcasing the relevant keywords, this study presents the key phrase extraction results obtained using the TextRank algorithm. Specifically, refer to Table 2 for the 15 top ranked key phrases.

**Table 2:** Ranking of key phrases extracted based on TextRank technology.

Rank	key phrase	weight
1	妇女儿童保护法Law on the Protection of Women and Children	0.49124711454168074
2	延长产假天数extended maternity leave days	0.4405755466548261
3	三姑六婆gossipy relatives	0.3274980763611205
4	丧偶式教育father-absent parenting	0.3274980763611205
5	短哺乳期short breastfeeding period	0.3274980763611205
6	婚育观attitudes towards marriage and fertility	0.3274980763611205
7	择偶观attitudes towards mate selection	0.3274980763611205
8	物以类聚人以群分Birds of a feather flock together	0.3274980763611205
9	祸害下一代harmful to the next generation	0.3274980763611205
10	纸片人Women are like paper dolls	0.3274980763611205
11	只生一个好It’s best to have only one child	0.11646999275731826
12	万里挑一对象finding a needle in a haystack	0.11646999275731826
13	快餐化婚姻fast food marriage	0.11646999275731826
14	上户口难Difficulty in obtaining household registration in rich cities	0.11646999275731826
15	耳濡目染的爱情love acquired through constant association	0.11646999275731826

Based on the extracted key phrases shown in the above table, certain themes were evident. Among them, the themes of “Women and Children Protection Law,” “Extension of Maternity Leave,” and “Short Breastfeeding Period” reflected concerns about women’s rights. The themes “Attitudes towards Marriage and Fertility” and “Mate Selection Criteria” indicated a focus on women’s perspectives on marriage and fertility. However, analysis based solely on a keyword table has limitations and may contain biases. Therefore, the key phrases extracted using the TextRank algorithm were abstracted and summarised into four dimensions to explore the factors influencing women’s marriage and fertility (Table 3).

**Table 3:** Abstract summary based on key phrase results.

Dimension	Key phrases
Social policies and rights protection	Law on the Protection of Women and Children
	extended maternity leave days
	short breastfeeding period
	Difficulty in obtaining household registration in rich cities
parenting concerns	father-absent parenting
	harmful to the next generation
Values and beliefs about marriage and fertility	attitudes towards marriage and fertility
	attitudes towards mate selection
	Birds of a feather flock together
	It’s best to have only one child
	fast food marriage
	finding a needle in a haystack
	love acquired through constant association
Family and social culture	gossipy relatives
	Women are like paper dolls

Based on the abstract summary in Table 3, the following results were inferred. First, based on the abstraction of 15 key phrases, the factors influencing negative sentiment orientation towards women’s marriage and fertility were categorised into four dimensions: “social policies and rights protection,” “parenting concerns,” “values and beliefs relating to marriage and fertility,” and “family and social culture.” Second, while additional factors or dimensions may influence negative emotional attitudes towards women’s marriage and fertility, the aforementioned four dimensions can be understood as the core factors determining these attitudes.

In conclusion, based on analysis of sentiment tendencies in Weibo comments, four key dimensions that contribute to the negative sentiment orientation towards women’s marriage and fertility in China were preliminarily identified. These dimensions comprise “social policies and rights protection,” “parenting concerns,” “values and beliefs related to marriage and fertility,” and “family and social culture.” These factors may have a negative impact on women’s attitudes and behaviours regarding marriage and fertility, leading to a prevailing negative sentiment. Addressing issues within these four dimensions can help redress the negative sentiment orientation towards women’s marriage and fertility in China, as well as addressing related issues such as low fertility rates and other interconnected challenges.

#### Mechanisms and pathways for transforming negative sentiment orientation towards women’s marriage and fertility

**Fig 11:**
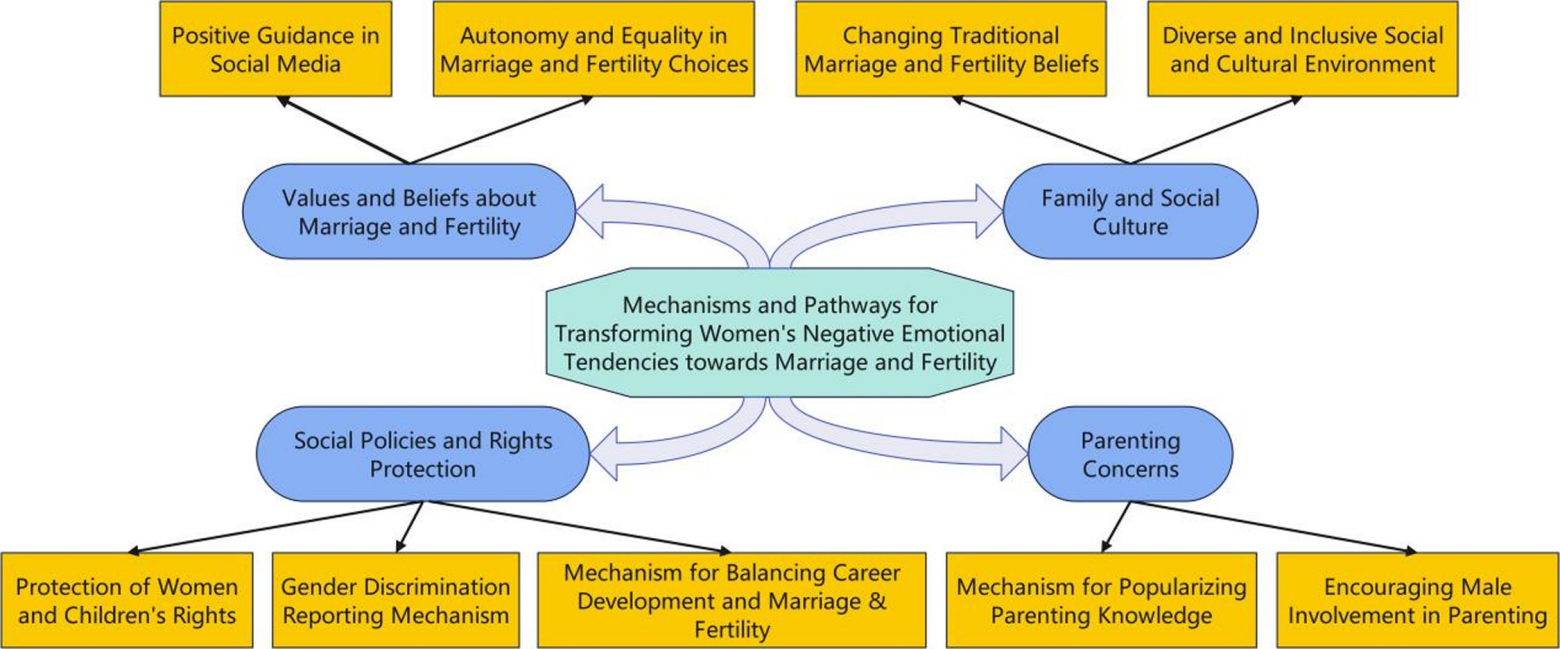
Mechanisms and pathways for transforming negative sentiment orientation towards women’s marriage and fertility.

The mechanisms and pathways for transforming negative emotional tendencies towards women’s marriage and childbirth are summarised and outlined in this study, as illustrated in Fig 11. The study’s findings indicate that four key dimensions influence women’s negative emotional tendencies regarding marriage and fertility, namely “social policies and rights protection,” “parenting concerns,” “values and beliefs about marriage and fertility,” and “family and social culture.”

## Discussion

This study found that the overall sentiment orientation towards six hot topics related to women’s marriage and fertility on Weibo was predominantly negative. This may have been due to the presence of implicit gender discrimination and inequality in the current socio-cultural context in China, which puts women under particularly great pressure in terms of marriage and fertility. Negative comments such as the following reflect the common experience of women facing job-related challenges: “It’s really ironic that every company in job interviews asks when you plan to have children, every single one! They would say that they think you have the ability, but they are concerned about having children in the future” [40, 41]. This leads to a negative orientation when discussing marriage and fertility. Additionally, media coverage and controversial remarks on social media can influence public emotions [42, 43], triggering discussion and arousing a negative sentiment orientation.

Based on sentiment evaluation of the comments on these six Weibo hot topics separately, we found that the majority of comments related to the following topics were negative: “The Marriage and Fertility Dilemma of Contemporary Young Women,” “Tendency of Some Young Women Towards Non-Marriage and Non-Childbearing,” and “Prohibition on Inquiring about Women’s Marriage and Fertility in Job Interviews”. First, concerning the difficulties faced by contemporary women in marriage and fertility, such negative sentiment may stem from societal expectations and pressure placed on women, whose primary roles are seen to relate to marriage and fertility, which may limit their pursuit of career and personal development [44–46]. Such negative sentiment is exemplified by comments such as “It’s like binding women’s feet again (foot binding was a cruel traditional practice in ancient China that involved tightly binding women’s feet to achieve a small and delicate foot shape, causing long-term physical and psychological harm to women.), and binding their minds as well, and then obediently becoming a baby-making machine, it’s laughable”. Furthermore, the inclination of some young women towards non-marriage and non-childbearing may evoke negative sentiment as it may be perceived as going against traditional family values [47, 48], sparking controversy and criticism. This was illustrated in our study by comments such as the following “In reality, many women passively get married because they fear isolation and marginalisation. All the women around you are talking about marriage. If you don’t get married, others will think you’re abnormal, even sick. Seriously, and if you don’t have huge wealth, don’t talk about being happy in old age with money”. Last, concerning the apparent prohibition on inquiring about women’s marital and fertility status during recruitment, negative sentiment may be associated with employment discrimination and gender bias [49–51]. Many people believe that such practices deprive women of their rights and equal opportunities. Such sentiment was illustrated in our study by comments such as the following: “We should advance women’s employment rights! Unmarried women are not hired, women with one child are not hired … Gender discrimination in the workplace is all too common. It’s the job-hunting season again, and the sacrifices women make for society should not result in unequal opportunities. Female employees should not have their wages reduced or be dismissed because of pregnancy, childbirth, or breastfeeding”.

The comments on the following topics had relatively positive emotion scores: “Why Are Women’s Topics Always Related to Marriage and Fertility?”, “How to View the Increasing Trend of Women’s Marriage and Fertility Delay” and “The Impact of App Usage Habits on College Students’ Attitudes towards Marriage and Fertility”. This may have been because such topics involve concern about and discussion of women’s marriage and childbearing in contemporary society, and positive emotions may reflect support for the concept of gender equality and the recognition of women’s right to choose independently [52]. Illustrative comments include “Break the prejudice, break the boundary, tear off the label, and live yourself”. Furthermore, discussion of Chinese women’s increasing tendency to delay marriage and childbearing and the influence of app usage habits on college students’ attitudes towards marriage and fertility may involve societal changes and the shifting values of the younger generation. Social media play a significant role in shaping the marriage and fertility values of college students [53, 54]. Positive sentiments expressed in comments may reflect an acceptance of societal changes and individual autonomy. Such comments include the following: “Of course, I will still get married. Even though I have seen the dark side of marriage through apps, I still have hope. Let’s not forget that there is a positive side to everything. There are many factors that lead to an unhappy ending! So, let’s strive to improve ourselves and become better (including our criteria for choosing a partner)”. In conclusion, the reasons for the positive sentiment in discussion of the aforementioned topics may be related to the recognition of gender equality, individual autonomy, and societal transformations.

Based on the results of our word cloud, word2Vec, and TextRank analyses, it is evident that the main factors influencing women’s negative sentiment towards marriage and fertility in China today are concerns about child-rearing, inadequate social policies and rights protection, shifting values regarding marriage and fertility, and the influence of traditional family and social cultural beliefs. First, concerns about child-rearing may arise from conflicts and contradictions between women’s pursuit of career development and personal freedom and the responsibilities of raising children [55, 56]. Mothers may worry about their ability to handle the challenges of parenting alone, including time, energy, and financial challenges. A lack of support from and involvement by fathers may also contribute to feelings of isolation and unfair treatment among mothers [57], thereby negatively impacting their emotional inclination towards marriage and fertility. Second, due to the lack of reasonable support and protection mechanisms in society, women may face further difficulties and receive unfair treatment relating to marriage and fertility. Additionally, exorbitant housing prices contribute to fear surrounding marriage and fertility [58, 59]. Shifting values regarding marriage and fertility may be influenced by modern societal notions, with women placing greater emphasis on personal desires and autonomous choices, leading to scepticism about and resistance to traditional views on marriage and fertility [60, 61]. Last, the influence of family and social cultural beliefs can result in women’s assuming a greater burden of household chores and childcare responsibilities, preventing the equal division of labour and support [62, 63] and thereby generating negative emotions. In conclusion, these factors collectively contribute to women’s negative emotions towards marriage and fertility, requiring further attention and exploration from relevant authorities.

Based on the results of this study, mechanisms and pathways for transforming women’s negative sentiments towards marriage and fertility are proposed, with the hope of providing guidance for relevant authorities responsible for policy making. First, it is recommended that government departments formulate and improve relevant social policies, including strengthening the protection of women’s and children’s rights, providing better support and welfare measures for childcare, and alleviating the economic burden and pressure on women in raising children. Second, efforts should be made to promote a shift in societal values, encouraging respect and support for women’s personal choices and career development, reducing traditional pressures and expectations regarding women’s marriage and fertility, and emphasising the autonomy and equality of marriage and fertility choices. Social media can play a role in guiding and shaping proper values related to marriage and fertility. Additionally, through educational and awareness campaigns, mechanisms for disseminating parenting knowledge should be enhanced, promoting active male involvement and responsibility in families to address the issue of absent fathers. Last, we recommend establishing mechanisms that support women’s career development and work–life balance, improve reporting systems for gender discrimination, including flexible work arrangements and career development paths, enabling women to better balance their family and professional lives and creating a more diverse and inclusive socio-cultural environment.

## Conclusion

This study identified a prevailing negative sentiment in Weibo discussions on women’s marriage and fertility, driven by societal pressures, discriminatory practices, and evolving gender roles. Topics related to contemporary challenges, changing attitudes among young women, and recruitment inquiries exhibited predominantly negative sentiments. However, discussions on broader women’s issues and societal trends garnered more positive responses. The study highlighted concerns about conflicting career and parenting expectations, inadequate societal support, and evolving values as major influencers of negative sentiments. To address these issues, recommendations include policy improvements, societal value shifts, positive social media influence, and enhanced support mechanisms for women’s career and family balance.

## Limitations

This study has some limitations. First, due to limited computational resources, the Word2Vec and TextRank models used for NLP in this study utilised only a small-scale pre-trained model, specifically designed for Chinese language processing. Second, the model did not consider the sentiment information contained in non-textual media that may appear in comments, such as images and emojis. Further research is needed to explore these aspects. Thirdly, this study did not revise the relevant experimental results of the State of the Art (SOTA) methods. Therefore, future research may consider revising the experimental results associated with SOTA methods to ensure that the study fully incorporates the latest methods and technologies in the field, thereby enhancing the quality and credibility of the research.

## Supporting information

S1 Data (XLSX)
